# Optimizing Immunotherapy: The Synergy of Immune Checkpoint Inhibitors with Artificial Intelligence in Melanoma Treatment

**DOI:** 10.3390/biom15040589

**Published:** 2025-04-16

**Authors:** Mohammad Saleem, Abigail E. Watson, Aisha Anwaar, Ahmad Omar Jasser, Nabiha Yusuf

**Affiliations:** 1 Department of Dermatology, Heersink School of Medicine, University of Alabama at Birmingham, Birmingham, AL 35294, USAaanwaar@uab.edu (A.A.); oj2@uab.edu (A.O.J.); 2 College of Medicine, Florida State University, Tallahassee, FL 32306, USA

**Keywords:** melanoma, immune checkpoint inhibitors, artificial intelligence, immunotherapy, PD-1, PD-L1

## Abstract

Immune checkpoint inhibitors (ICIs) have transformed melanoma treatment; however, predicting patient responses remains a significant challenge. This study reviews the potential of artificial intelligence (AI) to optimize ICI therapy in melanoma by integrating various diagnostic tools. Through a comprehensive literature review, we analyzed studies on AI applications in melanoma immunotherapy, focusing on predictive modeling, biomarker identification, and treatment response prediction. Key findings highlight the efficacy of AI in improving ICI outcomes. Machine learning models successfully identified prognostic cytokine signatures linked to nivolumab clearance. The combination of AI with RNAseq analysis had the potential for the development of personalized treatment with ICIs. A machine learning-based approach was able to assess the risk-benefit ratio for the prediction of immune-related adverse events (irAEs) using the electronic health record (EHR) data. Deep learning algorithms demonstrated high accuracy in tumor microenvironment analysis, including tumor region identification and lymphocyte detection. AI-assisted quantification of tumor-infiltrating lymphocytes (TILs) proved prognostically valuable in primary melanoma and predictive of anti-PD-1 therapy response in metastatic cases. Integrating multiple diagnostic modalities, such as CT imaging and laboratory data, modestly enhanced predictive performance for 1-year survival in advanced cancers treated with immunotherapy. These findings underscore the potential of AI-driven approaches to refine biomarker identification, treatment prediction, and patient stratification in melanoma immunotherapy. While promising, clinical validation and implementation challenges remain.

## 1. Introduction

Cancer remains a leading cause of morbidity and mortality globally, with projections indicating 2,001,140 new cases and 611,720 cancer deaths in the United States alone in 2024. The global cancer burden is expected to reach 28.4 million cases by 2040, a 47% increase from 2020 [1,2].

Melanoma, the deadliest form of skin cancer, accounts for approximately 1.7% of cancer diagnoses globally, and is a significant contributor to cancer-related mortality [3].

The encouraging trend of a declining mortality rate in the United States (1.3% annually between 1999 and 2020, with 184,416 melanoma-related deaths recorded) offers a glimmer of hope [4]. This improvement can be attributed, in part, to the advent and widespread adoption of immune checkpoint inhibitors (ICIs) as melanoma interventions [5]. However, despite this progress, a substantial number of patients still succumb to the disease, highlighting the ongoing need for optimized therapeutic approaches.

This review seeks to explore the intersection of ICIs and artificial intelligence (AI), with a particular focus on their applications in melanoma therapy. By critically evaluating the existing literature, we aim to elucidate how AI can refine ICI treatment strategies, improve predictions of immune responses, and ultimately contribute to better patient outcomes.

A comprehensive literature search was conducted using PubMed databases. The search strategy employed combinations of the following keywords and MeSH terms: (“immune checkpoint inhibitors” OR “anti-PD-1” OR “anti-PD-L1” OR “anti-CTLA-4”) AND (“melanoma”) AND (“artificial intelligence” OR “machine learning” OR “deep learning”). This review does not adhere to PRISMA guidelines, as it does not follow a systematic methodology for study selection, data extraction, or risk-of-bias assessment. Instead, it is a narrative review gathering relevant literature on the intersection of immune checkpoint inhibitors and artificial intelligence in melanoma treatment.

The inclusion criteria were limited to original research articles investigating the use of ICIs in melanoma treatment and/or the application of AI in predicting responses to ICIs. Studies focusing on other cancer types or immunotherapies were excluded, except in cases where they offered substantial insights relevant to melanoma treatment. Two independent reviewers screened titles and abstracts, followed by a full-text review of selected articles. Disagreements were resolved through discussion with a third reviewer.

## 2. Immune Checkpoint Inhibitors

Immune checkpoint inhibitors (ICIs) are a class of drugs that utilize antibodies to target and block specific immune checkpoint proteins [5]. Immune checkpoint proteins normally regulate immune tolerance; however, tumor cells exploit these checkpoints to evade immune surveillance and promote uncontrolled growth. ICIs target these proteins, modulating the immune response and enhancing the immune system’s ability to recognize and attack cancer cells [6]. ICIs have revolutionized cancer treatment by targeting regulatory proteins that suppress the immune system’s ability to detect and destroy cancer cells. Recent advances in ICIs hold promising potential to block these checkpoints. By overcoming this immune resistance, ICIs empower the immune system to recognize and target cancer cells more effectively, marking a significant advancement in cancer treatment [7].

ICIs represent a paradigm shift in cancer treatment, harnessing the power of the immune system to combat malignant cells [7]. These innovative therapies work by targeting specific immune checkpoint proteins, which normally act as regulators of immune tolerance, preventing excessive immune activation and autoimmunity [8]. However, cancer cells cleverly exploit these checkpoints to evade immune surveillance and proliferate unchecked [8]. ICIs, by blocking these inhibitory signals, effectively unleash the immune system’s cytotoxic potential against cancer [9]. This groundbreaking approach has revolutionized the treatment landscape for several cancer types, offering durable remissions and improved survival outcomes for many patients. However, not all patients benefit equally from ICI therapy, and resistance mechanisms remain a significant obstacle [9].

Within the realm of ICI therapy for melanoma, three primary classes of ICIs are utilized in melanoma treatment as summarized in Table 1. The first class includes anti-programmed cell death 1 (PD-1) inhibitors. PD-1 is a transmembrane receptor expressed on various immune cells, such as activated T and B cells, monocytes, dendritic cells, regulatory T cells, and natural killer T cells [10]. The PD-1 pathway, through its interaction with the ligands PD-L1 and PD-L2, exerts a critical regulatory role in T-cell activation, effector function, tolerance, and exhaustion. Its expression on T cells is particularly significant in the context of ICI therapy for melanoma. Therapeutic blockade of this pathway with clinically approved agents such as pembrolizumab, nivolumab, and cemiplimab has demonstrated significant efficacy in restoring anti-tumor immunity [9,10].

**Figure 1:**
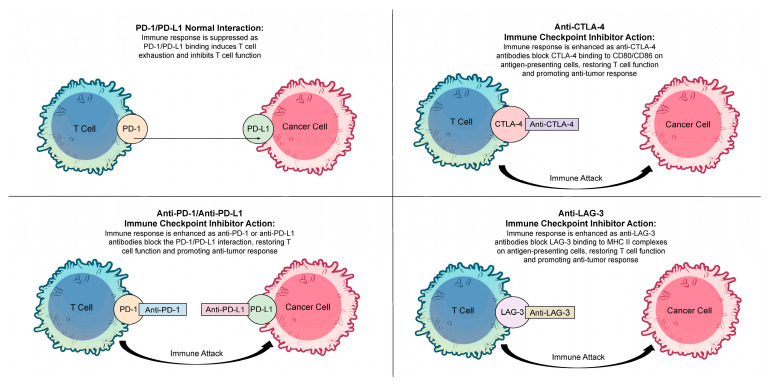
PD-1/PD-L1 Interaction and Immune Checkpoint Inhibitors.

The second class of ICIs targets PD-L1. PD-L1 is expressed in a variety of immune and non-immune cells, including antigen-presenting cells, activated T and B lymphocytes, and macrophages [11]. Within the tumor microenvironment, tumor cells exploit this pathway by upregulating PD-L1 expression in response to pro-inflammatory signals from activated T cells, thereby promoting immune evasion [12]. Therapeutic agents such as atezolizumab, avelumab, and durvalumab effectively disrupt this interaction, as PD-L1 binding to PD-1 on T cells suppresses T-cell activity and facilitates immune escape by tumor cells, as depicted in Figure 1 [13].

The third class of ICIs includes anti-CTLA-4 inhibitors. CTLA-4 is a co-inhibitory receptor expressed on activated T cells, serving as a pivotal regulator of the immune response [14]. It modulates the early stages of T-cell activation and maintains immune homeostasis by attenuating excessive inflammation. Importantly, CTLA-4 blockade not only enhances T-cell activation but has also been associated with an increase in the diversity of the peripheral T-cell repertoire in patients with melanoma [15]. This phenomenon, observed in recent studies, underscores the potential of CTLA-4 inhibitors to reshape the immune landscape and bolster anti-tumor immunity.

These ICIs work by blocking immune checkpoints, enhancing T-cell function, and amplifying the immune response against malignant tumors. In the TME, sustained high levels of PD-1 expression on T cells can lead to a dysfunctional state known as T-cell exhaustion. ICIs block PD-1/PD-L1 interactions, restoring T-cell functionality and promoting anti-tumor immune responses [16].

While these ICI therapies have significantly improved outcomes for some melanoma patients, challenges remain. A significant proportion of patients do not respond to monotherapy, and even those who initially benefit can develop resistance. Therefore, strategies to optimize ICI efficacy are crucial. One promising approach is the combination of anti-PD-1/PD-L1 and anti-CTLA-4 inhibitors, which has demonstrated synergistic anti-tumor activity in clinical trials [17]. This combined blockade targets two distinct immune checkpoints, potentially leading to a more comprehensive and sustained immune response. However, this combination is also associated with increased toxicity, highlighting the need for careful patient selection and management [17]. Furthermore, the optimal sequencing and dosing of these agents, as well as the identification of predictive biomarkers for response, are areas of ongoing research.

While ICIs have shown remarkable success, they can also trigger immune-related adverse events in ICI-treated individuals [18]. Additionally, cardiovascular toxicity is a relevant risk for patients treated with PD-1/PD-L1 inhibitors, including increased risk of hypertension, hypotension, myocarditis, and arrhythmia [18]. This has driven research into methods to personalize and refine cancer treatment, with artificial intelligence (AI) and machine learning (ML) emerging as powerful tools in this endeavor.

## 3. Artificial Intelligence and Prediction of Immune Responses with Immune Checkpoint Inhibitors

AI has emerged as a powerful tool for improving the prediction of immune responses and patient outcomes, with evidence demonstrating its ability to enhance accuracy and clinical decision-making in oncology. For instance, Wang et al. [19] developed a machine learning model capable of identifying prognostic cytokine signatures associated with nivolumab clearance in patients with advanced melanoma, paving the way for more personalized dosing strategies. Similarly, Johannet et al. [20] designed a deep convolutional neural network (DCNN) to analyze the tumor microenvironment in melanoma, offering novel insights into tumor-immune interactions. Koelzer et al. [21] advanced the field further by creating a supervised machine learning algorithm, specifically a Random Forest classifier, to enhance the precision of PD-L1 scoring in cutaneous melanoma.

The integration of AI with ICI therapy in melanoma treatment shows promising potential across multiple domains. Machine learning approaches have demonstrated significant capabilities in predicting treatment responses and identifying novel biomarkers for ICI therapy [22,23].

AI applications have shown particular promise in these key areas:

### 3.1. PD-L1 Expression Assessment

AI-based digital image analysis has improved the precision of PD-L1 scoring in cutaneous melanoma. Determination of PD-L1 expression by immunohistochemistry has become an important predictive biomarker across several cancers, including melanoma. Manual quantification of PD-L1 can lead to variation. AI-based approaches can mitigate the variation observed by manual assessment of PD-L1. Baxi et al. [24] compared the use of AI-powered algorithms with manual assessment and found more PD-L1 positive samples compared to manual scoring at cutoffs of ≥1% and ≥5% in several tumors, including melanoma. Similar improvements were seen in response and survival in patients who were PD-L1 positive patients compared to those who were PD-L1 negative.

In another study, a supervised machine learning algorithm showed high concordance with conventional pathologist assessments (Pearson’s correlation coefficient r = 0.97, *p* < 0.0001) [22]. This suggests that AI-based scoring systems could standardize PD-L1 assessment and potentially reduce inter-observer variability in clinical practice, leading to more consistent and reliable treatment decisions [22].

### 3.2. Assessment of Resistance to Immune Checkpoint Inhibitors

Interactions of cells in the tumor microenvironment (TME) are important for the growth of tumors and clinical outcomes. ICI therapy is based on ligand-receptor interaction. Characterization of these interactions is an integral part of predicting resistance to ICIs, and for the development of novel drug targets. Resistance to ICIs can be classified as innate (pre-existing) or acquired (developing during therapy). Innate resistance refers to the tumor’s inherent ability to evade immune detection, often due to immunosuppressive factors within the TME or alterations in immune checkpoints. Acquired resistance, on the other hand, occurs after initial responsiveness to ICIs, when tumors adapt and develop mechanisms to escape immune surveillance over time. To delineate bulk transcriptomics and cell-cell interactions, Sahni et al. [25] developed COnfident DEconvolution For All Cell Subsets (CODEFACS) and LIgand-Receptor Interactions between Cell Subsets (LIRICS), respectively. On the basis of these methods, they developed a supervised machine learning model, Immunotherapy Resistance cell-cell Interaction Scanner (IRIS) to detect cell-type specific TME ligand-receptor interactions that were relevant to resistance acquired after treatment with ICIs. Using this model to transcriptomics data of five melanoma cohorts, they were able to identify specific resistance downregulated interactions (RDIs) in ICI therapy-resistant tumors. These TME assessments, specifically identifying key ligand-receptor interactions associated with resistance, can help select the most appropriate ICI therapy for individual patients. By understanding the specific interactions within the TME that drive resistance, clinicians may tailor therapies to target these pathways more effectively, potentially improving patient responses and overcoming resistance to ICIs

### 3.3. Clinical Management of Immune-Related Adverse Events

Treatment with ICIs often comes with the risk of immune-related adverse events (irAEs) in patients with melanoma. The most common ones are often dermatologic, endocrine, gastrointextinal, and hepatic. Some rare types of neurologic, urologic, pulmonary, and cardiac outcomes can also occur. Since ICIs are used in common practice, it is important to understand the irAEs in the general population, especially in older adults, who comprise a majority of the population with advanced melanoma [26].

Prediction of irAE risk can provide a personalized risk-benefit profile. Lippenszky et al. [27] developed a machine learning framework using electronic health record (EHR) data to predict irAEs and 1-year overall survival. A total of 37.8% of patients had melanoma and the cohort was predominantly male. Their model showed strong performance with an AUC of 0.739. This is the first reported machine learning-based solution for the assessment of the risk-benefit ratio using the EHR data.

Artificial intelligence chatbots are being extensively used for general and medical information but their accuracy is questionable. Burnette et al. [28] developed questions and answers in guidelines surrounding 10 irAE categories and an additional 20 patient scenarios. These scenarios included questions such as which patient populations should not receive ICIs, the main treatments for grade 3 or higher ICI toxicities (including medication doses and duration), major complications of high-dose steroids, the surveillance labs that should be obtained in all patients treated with ICIs, and the risks of using combination immune checkpoint inhibition compared to anti-PD-1 monotherapy, and many more. They questioned two AI chatbots (ChatGPT (V.GPT-4) and Bard 2.0). The accuracy of responses was assessed by experts in irAE management using a Likert scale, and answers were compared across categories and engines. They found that ChatGPT provided generally more accurate information and detailed responses, but some errors were still noted. Additional verification may be needed after the use of this modality.

### 3.4. Deep Learning Applications in Treatment Response Prediction

Recent studies have explored the use of deep learning models for predicting immunotherapy response. One notable study utilizing whole-slide images to predict anti-PD-1 response in melanoma patients achieved an AUC of 0.778 in melanoma testing samples, correctly classifying 65.2% of responders and 74.2% of non-responders [29]. This performance was superior to conventional TIL assessment (AUC 0.58), suggesting that deep learning approaches may offer improved predictive capabilities for ICI response in melanoma patients, allowing for earlier identification of non-responders and exploration of alternative treatment strategies [30].

Deep convolutional neural network (DCNN) classifiers have achieved remarkable accuracy in identifying key tissue components, including tumor regions (AUC 0.961), lymphocytes (AUC 0.962), and connective tissue (AUC 0.969) [20]. This precise characterization of the tumor microenvironment is crucial for understanding ICI response patterns and could potentially identify patients who are more likely to benefit from specific ICI therapies. These advancements in digital pathology, driven by AI, offer the potential to move beyond traditional histopathological assessments and provide more objective and quantitative measures for clinical decision-making.

The study by Chatziioannou et al. [31] provides compelling evidence for the prognostic and predictive value of AI-assisted tumor-infiltrating lymphocyte (TIL) quantification in melanoma. To begin with, the study emphasizes the predictive potential of AI-assisted TIL quantification. Using a deep learning algorithm (NN192), they developed an electronic TIL score (eTILs) that demonstrated significant prognostic value in primary melanoma and predictive potential for anti-PD-1 therapy response in metastatic disease. For metastatic melanoma, eTILs > 12.2% in therapy-naïve samples correlated with improved progression-free survival (*p* = 0.037) and melanoma-specific survival (*p* = 0.0038) in patients receiving anti-PD-1-based immunotherapy [31]. These findings suggest that AI-assisted TIL quantification could serve as a predictive biomarker for immunotherapy response in advanced disease, potentially guiding treatment decisions and improving patient outcomes. Furthermore, the study also highlights the assessment capabilities of AI-assisted TIL quantification. In primary melanomas, an eTILs ≤ 16.6% was associated with unfavorable relapse-free survival (*p* = 0.0012) and remained significant in multiple Cox regression (*p* = 0.0161), complementing current staging methods [31]. This underscores the potential of AI to refine traditional histopathological assessments and provide more objective and quantitative measures for clinical decision-making, helping with risk stratification in early-stage melanoma.

Another promising application of AI in melanoma immunotherapy is the development of personalized dosing strategies based on prognostic cytokine signatures. The study by Wang et al. [19] demonstrates the potential of machine learning in identifying prognostic cytokine signatures associated with nivolumab clearance in patients with advanced melanoma. Using a random forest algorithm, they developed a model that selected 16 top-ranking baseline inflammatory cytokines to predict nivolumab clearance. This model achieved an area under the curve (AUC) of 0.75 and an accuracy of 0.7 in classifying patients into high and low-clearance groups. Importantly, the predicted clearance (high vs. low) based on this cytokine signature was significantly associated with overall survival across three phase III studies (*p* < 0.01), regardless of treatment (nivolumab vs. chemotherapy). This research highlights the potential of using AI-derived cytokine signatures to stratify patients based on their likelihood of response to nivolumab treatment, potentially enabling more personalized therapeutic approaches in advanced melanoma [19]. This research highlights the potential of using AI-derived cytokine signatures to stratify patients based on their likelihood of response to nivolumab treatment, potentially enabling more personalized therapeutic approaches to advanced melanoma. This demonstrates the power of AI to integrate complex biological data (cytokines) with clinical outcomes (survival) to identify predictive biomarkers.

The integration of multiple diagnostic modalities, including imaging, laboratory data, and clinical parameters, has the potential to significantly improve prognostic accuracy in melanoma patients treated with immunotherapy. The study by Yeghaian et al. [32] investigated the integration of multiple noninvasive diagnostic modalities to predict 1-year survival in patients with advanced non-small-cell lung cancer, melanoma, and urothelial cancer treated with immunotherapy. Using an ensemble of AI models trained on chest CT imaging, routine laboratory blood tests, and clinical parameters, they demonstrated that integrating different diagnostic data modalities modestly improved predictive performance. The highest area under the curve (AUC) of 0.83 was achieved by combining CT and laboratory data, compared to AUCs of 0.81 and 0.73 for laboratory and CT data alone, respectively. The study included 475 patients with longitudinal data and utilized a model-agnostic late fusion strategy for multimodal integration. This research highlights the potential of AI-driven multimodal data integration in enhancing prognostic accuracy for cancer patients undergoing immunotherapy, potentially enabling more personalized treatment approaches [32]. The study by Yeghaian et al. [32] investigated the integration of multiple noninvasive diagnostic modalities in predicting survival in patients with advanced cancers, including melanoma, treated with immunotherapy. While this study examined multiple cancer types, its findings on multimodal data integration are relevant to melanoma as they demonstrate the potential for combining imaging, lab data, and clinical parameters to improve prognostic accuracy. This underscores the importance of a holistic approach to patient assessment, leveraging AI to integrate diverse data sources for more comprehensive risk stratification.

The study by Luo et al. [33] first used machine learning methods to construct an immunotherapy resistance score (TSIRS) based on tumor neoantigen burden (TNB) and cancer stemness and subsequently investigated the efficacy of the TSIRS in predicting outcomes of patients undergoing anti-PD1/PDL1 therapy. The ability of the TSIRS to predict the characteristics of tumor microenvironment during anti-PD1/PDL1 therapy was also investigated. Using multi-omics data and clinic information from various anti-PD1/PDL1 therapy cohorts, 26 gene modules correlated to TNB and cancer stemness were identified using a collection of bioinformatics methods. The module “MElightgreen” was significantly correlated with TNB and therapy responsiveness. The “MElightgreen” module refers to a specific gene expression module identified through the analysis of transcriptomics data, consisting of co-expressed genes that may be linked to specific biological processes or disease characteristics. Genes in the “MElightgreen” module to be used for the construction of the TSIRS were selected and their coefficients were calculated using machine learning-based methods, with the sum of the product of gene expression and coefficient determined as the TSIRS. The TSIRS of the training cohort was calculated, and the cohort was divided into high and low TSIRS groups. The study found that prognosis and drug responsiveness were worse in the high TSIRS group. The study found that the immunogenicity of tumors was inversely proportional to TSIRS. The study also found that PD-1 and PD-L1 expressions were also inversely proportional to TSIRS. The study also calculated the TSIRS of samples in two cohorts, a melanoma immunotherapy cohort and a clear cell renal cell carcinoma immunotherapy cohort. In both external validation cohorts, a high TSIRS correlated with significantly worse prognosis and lower therapy responsiveness, suggesting the high efficacy of the TSIRS in predicting outcomes of patients undergoing anti-PD1/PDL1 therapy [33]. Although the purpose of this study was to use machine learning methods to develop an immunotherapy resistance score to predict anti-PD1/PD-L1 response in all types of cancers, the results of the study are particularly relevant to melanoma as one of the two external validation cohorts used was a melanoma immunotherapy cohort. This demonstrates the potential of machine learning in helping develop predictive models that can be used to personalize cancer treatment in patients.

These advancements in AI applications for ICI therapy in melanoma demonstrate the potential for more personalized and effective treatment strategies. However, realizing this potential requires addressing several key challenges. Data quality and standardization are paramount, as AI models are only as good as the data they are trained on. Model interpretability is also crucial for clinical adoption. Clinicians need to understand *how* AI models arrive at their predictions to trust and effectively use them [34]. Regulatory considerations regarding the validation and approval of AI-based diagnostic and prognostic tools must also be addressed to ensure patient safety and efficacy. Future research should prioritize large-scale, prospective clinical trials to validate the efficacy of AI-driven decision-support tools in real-world clinical settings. This will provide the robust evidence needed for widespread clinical adoption and inform regulatory frameworks. Furthermore, the development of explainable AI (XAI) techniques is essential for enhancing clinical trust and facilitating integration into clinical workflows [35]. By addressing these challenges through collaborative efforts between clinicians, data scientists, and regulatory bodies, the integration of AI with ICI therapy has the potential to transform melanoma care, ushering in a new era of precision immunotherapy and significantly improving patient outcomes.

A deep learning model based on baseline computed tomography (CT) of melanoma lesions was unable to predict ICI response in advanced melanoma [36]. The limitation was the use of a single CT image of melanoma lesions. Spectral CT imaging or metrics of body composition from baseline CT imaging may provide more accurate information about the ICI response in patients with melanoma. A CT-based body composition study in patients on ICIs for metastatic melanoma, using deep learning, revealed that lower skeletal muscle index was associated with worse outcomes [37]. Serum lactate dehydrogenase (LDH) is an important biomarker for response to ICIs but it cannot differentiate between pseudo- and true progression. Tabari et al. [38] assessed the role of machine learning radiomics in predicting the response to ICIs in combination with LDH for the prognosis of metastatic melanoma. They found that individual radiomics and LDH models performed in a similar manner for the prediction of response. However, the combination of machine learning-based models was able to make more precise and individualized treatment decisions.

## 4. Conclusions

The convergence of ICI therapy and AI holds immense promise for transforming melanoma treatment. AI-driven approaches have demonstrated potential in enhancing biomarker identification, treatment response prediction, and digital pathology analysis, leading to more precise risk stratification and treatment selection. However, realizing the full potential of this integration requires addressing several key challenges. Data integration across diverse datasets and the subsequent standardization of AI-based tools, rigorous clinical validation through prospective trials, and the development of explainable AI (XAI) techniques to enhance clinical trust and adoption are crucial steps. Future research should prioritize prospective clinical trials to directly evaluate the clinical utility and cost-effectiveness of AI-driven decision-support tools in real-world settings. This will not only provide the robust evidence needed for widespread clinical adoption and inform regulatory frameworks but also pave the way for equitable access to these innovative technologies. By fostering collaboration between clinicians, data scientists, and regulatory bodies to overcome these challenges, the integration of AI with ICI therapy has the potential to redefine melanoma care and usher in a new era of precision immunotherapy.

With continued research and collaborative efforts between clinicians, data scientists, and regulatory bodies, the integration of AI with ICI therapy has the potential to significantly improve patient outcomes and reshape the landscape of melanoma treatment.

## Figures and Tables

**Table 1:** FDA-Approved Immune Checkpoint Inhibitors for Melanoma Treatment.

Drug Name (Brand Name)	Target	Type	Initial FDA Approval	Key Indications for Melanoma	Common Side Effects
Pembrolizumab (Keytruda)	PD-1	Monoclonal Antibody	2014	Unresectable or metastatic melanoma; adjuvant treatment of melanoma with involvement of lymph node(s) following complete resection	Fatigue, rash, diarrhea, pruritus, nausea, arthralgia, immune-mediated adverse events (e.g., colitis, pneumonitis)
Nivolumab (Opdivo)	PD-1	Monoclonal Antibody	2014	Unresectable or metastatic melanoma; adjuvant treatment of melanoma with involvement of lymph node(s) following complete resection	Fatigue, rash, diarrhea, pruritus, nausea, arthralgia, immune-mediated adverse events (e.g., colitis, pneumonitis)
Atezolizumab (Tecentriq)	PD-L1	Monoclonal Antibody	2016	Atezolizumab is not typically used as a single agent for melanoma. It is sometimes used in combination with other therapies in clinical trials.	Fatigue, nausea, decreased appetite, diarrhea, immune-mediated adverse events such as hepatitis, pneumonitis.
Avelumab (Bavencio)	PD-L1	Monoclonal Antibody	2017	Avelumab is not typically used as a single agent for melanoma. It has been investigated in combination with other therapies in clinical trials.	Fatigue, infusion-related reactions, diarrhea, immune-mediated adverse events.
Durvalumab (Imfinzi)	PD-L1	Monoclonal Antibody	2017	Durvalumab is not typically used as a single agent for melanoma. It has been investigated in combination with other therapies in clinical trials.	Fatigue, cough, nausea, immune-mediated adverse events.
Ipilimumab (Yervoy)	CTLA-4	Monoclonal Antibody	2011	Unresectable or metastatic melanoma; adjuvant treatment of melanoma with involvement of lymph node(s) following complete resection	Fatigue, diarrhea, pruritus, rash, immune-mediated adverse events (e.g., colitis, hepatitis, endocrinopathies).
Tremelimumab (I judo)	CTLA-4	Monoclonal Antibody	2022	In combination with durvalumab for unresectable hepatocellular carcinoma. It is not approved as a monotherapy for melanoma.	Fatigue, diarrhea, rash, decreased appetite, immune-mediated adverse events.
Relatlimab/Nivolumab (Opdualag)	LAG-3/PD-1	Dual Monoclonal Antibody	2022	Unresectable or metastatic melanoma	Fatigue, musculoskeletal pain, rash, pruritus, diarrhea, nausea, decreased appetite, immune-mediated adverse events

## Data Availability

Not applicable.
